# On the Use and Abuse of Filing to Remove Irregularities of the Teeth

**Published:** 1844-01

**Authors:** 


*On the Use and Abuse of Filing to remove Irregularities of the Teeth.*—There are many instances in which it is advisable and safe, as well as ornamental, to file the teeth; but as it is usually practised, nothing can be more pernicious.

Since it cannot be supposed that any man is so lost to shame and humanity as to expose his patient to pain and inconvenience during life, merely for the sake of a trifling fee, the indiscriminate filing of teeth, so common at present, should be imputed only to ignorance, and may, I hope, be checked, by placing the subject in a clear light, and by drawing the line to distinguish where it may, and where it may not, be practised with safety.

1. In people far advanced in years, the teeth may be filed into order without any inconvenience; because the nerves are lost, the teeth can feel no pain in the operation, nor afterwards from colds, acids, or sweets, and because they are not then so much disposed to caries or decay.

2. Where a tooth projects beyond the common level, and hinders the rest from meeting equally, or receives, on itself alone, all the pressure which should fall divided on a whole set, there filing is necessary, at any age, to remove the greater evil.

3. Filing is necessary and advisable to remove sharp points occasioned by fracture or otherwise, which irritate and wound the lips and tongue; because, in this case, the bony part of the tooth is already exposed, and cutting off the sharp prominences cannot make it more liable to caries or pain than it would otherwise have been.

4. Where a tooth points obliquely against the tongue, or against the lips, as often happens on account of the resistance of the milk-teeth, it is necessary to round the edge by filing, to prevent its wounding the soft parts.

5. When the edges of the fore teeth are uncommonly sharp and thin, and therefore apt to splinter, it is very proper to file them down, to give them a more obtuse and durable edge.

6. Filing is likewise advisable to remove caries, to prepare a tooth for the reception of a new crown, and in a few similar cases, related in the second part of this treatise.

7. When the teeth stand irregularly, and are too broad to admit of being reduced to one uniform line, filing between them may be practised to a certain degree; but great care should be taken not to cut away the enamel totally, as is too often practised on this occasion.

If a man had no feeling, nor any other use for his teeth but for the ornament of his countenance, I should not limit the use of the file to these cases only. But since most people, from infancy to middle age, feel insufferable pain the very moment the file touches the bony substance, and since this pain must be very often repeated, because each successive surface of the osseous substance must have some time to wither, and lose a part of its sensibility before it can admit of filing beyond a certain depth; since it happens, likewise, that the enamel once removed, is never regenerated; that a tooth in this naked state is for a long time affected with pain from the slightest impressions of cold, acids, sweets, &c.; that it wears away quickly, and is very much subject to decay, I cannot join to support the common practice of indiscriminate filing; I think it should be confined to the cases above related, for I believe it is advisable in these only.

Hence it is, that I so frequently refuse to perform this ornamental operation for my patients, and that I have often advised young people, who have credulously listened to advertisements and promises of this kind, never to barter a sure and valuable blessing for such a painful, dangerous, short-lived ornament: for ill-set or irregular teeth may last healthy and unpaired to the latest period of life, and the deformity in general is not very great, provided they are kept clean, white, and polished.—*Berdmore's Treatise on the Disorders and Deformities of the Teeth and Gums.*

